# The DAG1 transcription factor negatively regulates the seed-to-seedling transition in Arabidopsis acting on ABA and GA levels

**DOI:** 10.1186/s12870-016-0890-5

**Published:** 2016-09-09

**Authors:** Alessandra Boccaccini, Riccardo Lorrai, Veronica Ruta, Anne Frey, Stephanie Mercey-Boutet, Annie Marion-Poll, Danuše Tarkowská, Miroslav Strnad, Paolo Costantino, Paola Vittorioso

**Affiliations:** 1 Istituto Pasteur Italia - Fondazione Cenci Bolognetti, Rome, Italy; 2 Dipartimento di Biologia e Biotecnologie “C. Darwin”, Sapienza Università di Roma, Piazzale Aldo Moro 5, 00185 Rome, Italy; 3 Institut Jean-Pierre Bourgin, UMR1318, INRA, AgroParisTech, Université Paris-Saclay, RD10, 78026 Versailles, Cedex France; 4 Laboratory of Growth Regulators, Centre of the Region Haná for Biotechnological and Agricultural Research, Institute of Experimental Botany ASCR & Palacký University, Šlechtitelů 11, CZ-783 71 Olomouc, Czech Republic

**Keywords:** DAG1, Seed development, Chromatin remodelling, GA, ABA, *Arabidopsis thaliana*, DOF proteins

## Abstract

**Background:**

In seeds, the transition from dormancy to germination is regulated by abscisic acid (ABA) and gibberellins (GAs), and involves chromatin remodelling. Particularly, the repressive mark H3K27 trimethylation (H3K27me3) has been shown to target many master regulators of this transition. DAG1 (DOF AFFECTING GERMINATION1), is a negative regulator of seed germination in *Arabidopsis*, and directly represses the GA biosynthetic gene *GA3ox1 (gibberellin 3-β-dioxygenase 1*). We set to investigate the role of DAG1 in seed dormancy and maturation with respect to epigenetic and hormonal control.

**Results:**

We show that *DAG1* expression is controlled at the epigenetic level through the H3K27me3 mark during the seed-to-seedling transition, and that *DAG1* directly represses also the ABA catabolic gene *CYP707A2*; consistently, the ABA level is lower while the GA level is higher in *dag1* mutant seeds. Furthermore, both DAG1 expression and protein stability are controlled by GAs.

**Conclusions:**

Our results point to DAG1 as a key player in the control of the developmental switch between seed dormancy and germination.

**Electronic supplementary material:**

The online version of this article (doi:10.1186/s12870-016-0890-5) contains supplementary material, which is available to authorized users.

## Background

The transition from a growth-arrested seed to a germinating seed represents a crucial developmental switch in the life cycle of a plant [1]. Seeds of several annuals, including Arabidopsis, develop dormancy during the late stages of their development: although mature, these seeds are not capable of germinating even under favourable environmental conditions. Indeed, seed dormancy has been crucial for adaptation and evolution of seed plants.

Development of the Arabidopsis embryo consists of two phases: embryogenesis (from 0 to 6 days after pollination, DAP), and the embryo growth phase (from 7 to 10 DAP). Subsequently, seed maturation takes place until 21 DAP, when the seed is fully developed. Dormancy is established once embryo development is completed [2], and it is released within few weeks to several months after seed harvest, depending on the ecotype [3, 4].

Abscisic acid (ABA) produced during seed maturation is necessary to induce seed dormancy; gibberellins (GAs) release dormancy and promote germination, thus counteracting the effects of ABA, whereas the role of GAs during seed development is less clear. It has been shown that the increase in ABA level is crucial for proper progression through maturation, and a high ratio of ABA to GAs is the main determinant for the establishment of dormancy [3, 5].

While the importance of the dynamic balance between ABA and GAs is clear, so far the molecular mechanisms underlying seed dormancy induction, maintenance and release remain poorly understood [6].

Genetic analysis allowed the identification of a number of seed dormancy regulatory factors. Among these, DOG1 (DELAY OF GERMINATION 1), isolated by Quantitative Trait Loci (QTL) analysis, has been identified as a “seed-dormancy-specific” factor [7–9]. Although the genetic role of DOG1 has been well described, its molecular function remains still unknown.

The involvement of an epigenetic control in seed dormancy and germination has been recently proposed [10]. Indeed, the *DOG1* gene is marked by H3K27me3, a repressive epigenetic trait, and is upregulated upon loss of Polycomb Repressive Complex 2 (PRC2), responsible for this epigenetic mark [11]. PRC2 is required for the switch from embryonic to vegetative growth, and seeds lacking a functional PRC2 showed enhanced dormancy and germination defects [11].

We have previously shown that inactivation of the gene *DAG1* (*DOF AFFECTING GERMINATION1*) reduces seed dormancy [12]. DAG1 is a repressor of the seed germination process in Arabidopsis: *dag1* null mutant seeds require lower GAs and red light fluence rates than wild type seeds to germinate [12–14].

We have also demonstrated that DAG1 acts in the seed germination phytochromeB (phyB)-mediated pathway, downstream of PIL5 (PHYTOCHROME INTERACTING FACTOR3 LIKE5), and it negatively regulates the GA biosynthetic gene *GA3ox1*, by directly binding to its promoter [15, 16]. In addition, inactivation of *DAG1* results in an increase of the ABA catabolic gene *CYP707A2* in germinating mutant seeds, suggesting that DAG1 may regulate this gene [15].

More recently, we showed that the DELLA protein GAI (GA INSENSITIVE) interacts with DAG1 thus cooperating in repressing *GA3ox1* [16].

In the present study, we point to a key role of DAG1 in the developmental switch between seed dormancy and germination, and in the seed-to-seedling transition process. Indeed, DAG1 controls the level of GAs and ABA during seed maturation and dormancy by repressing *GA3ox1* and *CYP707A2* through direct binding to their promoters. Consistently, in *dag1* mutant seeds the ABA level is reduced while the level of GAs is increased. In addition, our data show that GAs control *DAG1* expression and DAG1 protein stability during imbibition. Furthermore, we show that the expression profile of *DAG1* is controlled at the epigenetic level through the H3K27me3 repressive mark, which is known to target regulatory genes of the seed-to-seedling stage.

## Results

### *DAG1* is expressed during seed maturation and dormancy and is modulated via epigenetic control

We have previously shown that inactivation of *DAG1* reduces seed dormancy [12]. To assess whether and when DAG1 is involved in the establishment of dormancy, we analysed its expression from late-maturation to non-dormant wild type seeds (developing seeds dissected from siliques at 13, 16, and 19 days after pollination, DAP, and dry seeds at 0 and 28 days after harvest, DAH) by means of RT-qPCR.

**Fig. 1:**
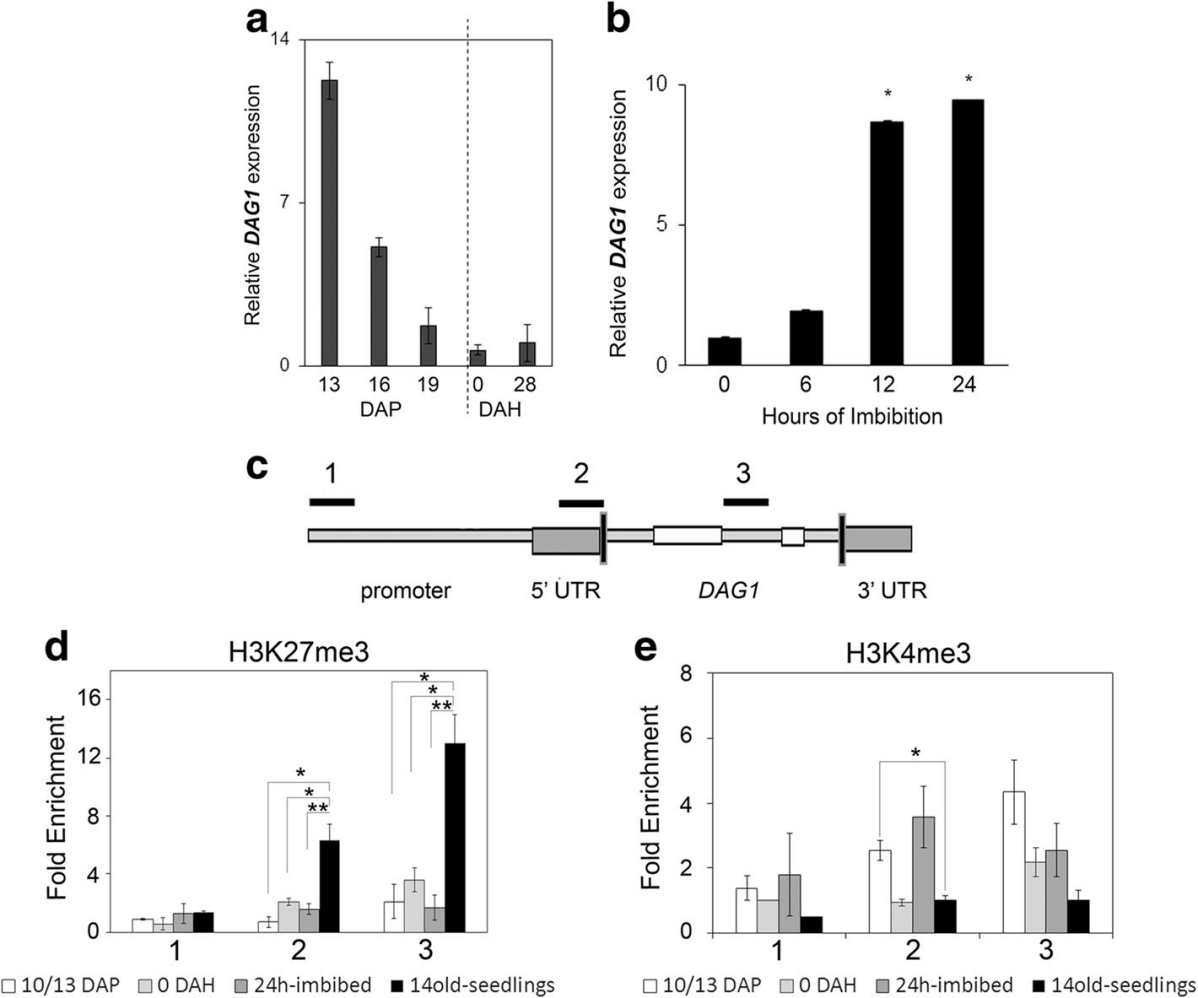
*DAG1* expression profile is controlled at epigenetic level. **a** Relative expression level of *DAG1* in wild type (WT) developing seeds at 13, 16 and 19 days after pollination (DAP), and in mature dry seeds at 0 and 28 days after harvest (DAH). **b** Relative expression level of *DAG1* in WT seeds, after 0, 6, 12 and 24 h imbibition. The values of relative expression levels are the mean of three biological replicates, presented with SD values. Significant differences were analyzed by *t*-test (*P ≤ 0,05). Expression levels were normalized with that of the *UBQ10* (*At4g05320*) reference gene. **c** Graphic representation of the *DAG1* locus. Above thick lines marked by numbers (1, 2, 3) are referred to different regions used for qPCR, corresponding to *DAG1* promoter (1), 5′end (2) and coding sequence (3) respectively. **d** and **e** Chromatin from WT developing seeds at 10/13 DAP, 0 DAH, 24 h-imbibed seeds, and 14 days-old seedlings was immunoprecipitated with specific antibodies against the H3K27me3 (**d**), or the H3K4me3 (**e**) epigenetic marks. The amount of DNA was measured by qPCR. The values of fold enrichment were normalized to internal controls (relative to input and to PP2A), and are the average of three biological replicates presented with SD values. Significant differences were analyzed by *t*-test (*P ≤ 0,05), and calculated with respect to 0 DAH

This analysis revealed that *DAG1* is highly expressed at 13 DAP, and that its expression subsequently decreases (16 DAP) to reach at 19 DAP a steady low level that is retained during dry storage (Fig. 1a).

*De novo* RNA synthesis is rapidly induced in non-dormant seeds following imbibition [17]: we therefore analysed *DAG1* expression in seeds imbibed for 6, 12 and 24 h, compared to dry seeds. As shown in Fig. 1b, the *DAG1* transcript level strongly increased following imbibition, reaching after 24 h a level almost 10-fold that of dry seeds.

Genome-wide studies revealed that genes mainly expressed in seeds are controlled at the epigenetic level through the H3K27me3 repressive mark in seedlings [11]. This prompted us to analyse the H3K27me3 profile of *DAG1* at different seed developmental stages - maturation (10/13 DAP), dormancy (0 DAH) and germination (24 h imbibed seeds) - and also in 14 days-old seedlings, similarly to Bouyer et al. [11]. We measured the enrichment of H3K27me3 by chromatin immunoprecipitation (ChIP) with specific antibodies against H3K27me3, or without antibodies as a negative control (Additional file 1: Figure S1), followed by quantitative PCR (qPCR) of three regions of the *DAG1* locus: a region of the promoter (1), one in the 5′ end (2) and one in the transcribed region (3) (Fig. 1c). Interestingly, the levels of H3K27me3 were significantly higher only in seedlings compared to the other three developmental stages, in regions 2 and 3 (Fig. 1d), consistent with the notion that the H3K27me3 epigenetic mark is usually restricted to the transcribed regions of target genes [18].

Since it is known that chromatin dynamics of several regulatory genes make use of two antagonistic marks, namely the repressive mark H3K27me3 and the activating mark H3K4me3, we verified whether *DAG1* also bears H3K4me3, to confirm that its expression may be regulated by dynamic changes in H3 methylation. This analysis, performed with H3K4me3 specific antibodies and without antibodies as a negative control (Additional file 1: Figure S1), clearly revealed a significant enrichment of the H3K4me3 activating mark during seed maturation in developing seeds at 10/13 DAP compared to seedlings, in the transcribed region of the *DAG1* gene (region 2) (Fig. 1e), consistent with the high level of *DAG1* expression during this stage (Fig. 1a and b).

### DAG1 acts on ABA and GA metabolism to establish and maintain seed dormancy

Genetic studies indicate that the control of seed dormancy and germination is the result of the balance between the levels of ABA and GA [19]. Since we had previously shown that inactivation of *DAG1* affects the expression of both the ABA catabolic gene *CYP707A2* and of the GA biosynthetic gene *GA3ox1* in germinating seeds [15], we extended the analysis by measuring by RT-qPCR the expression of the main ABA and GA metabolic genes - the catabolic gene *CYP707A2* encoding the ABA 8′-hydroxylase*,* the biosynthetic genes *NCED6* and *NCED9* encoding the 9-*cis*-epoxycarotenoid dioxygenase for ABA, the catabolic gene *GA2ox1* encoding GA2-oxidase, the biosynthetic genes *GA3ox2* and *GA3ox1* encoding GA3-oxidases for GA - in *dag1* and wild type developing seeds at 13, 16, 19 and 21 DAP encompassing the mid to late embryo maturation stages.

**Fig. 2:**
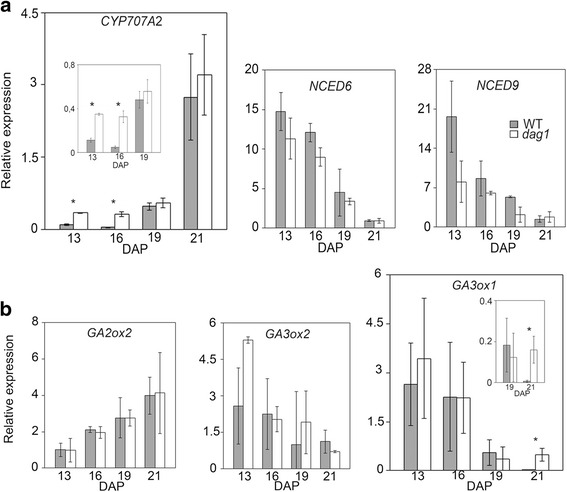
DAG1 controls ABA and GA pathways during maturation of seeds Relative expression level of: **a** *CYP707A2*, *NCED6* and *NCED9*, **b** *GA3ox1*, *GA3ox2* and *GA2ox2*, in *dag1* and wild type (WT) developing seeds at 13, 16, 19 and 21 DAP. The inset shows a magnification of the significant points. The values of relative expression levels are the mean of three biological repeats, presented with SD values. Expression levels were normalized with that of the *UBQ10* (*At4g05320*) gene. Significant differences were analyzed by *t*-test (*P ≤ 0,05)

As shown in Fig. 2a, of the ABA-related genes only the expression of *CYP707A2* was increased in *dag1* developing seeds compared to the wild type at 13 and 16 DAP (3.5- and 8-fold, respectively), but not at later maturation stages (19-21 DAP).

As for GA, the expression of *GA3ox1* was comparable in *dag1* and wild type developing seeds at 13 and 16 DAP, and also at 19 DAP when it dropped sharply in both stages of developing seeds, whereas at 21 DAP it was more than 26 times higher in *dag1* than in wild type developing seeds (Fig. 2b).

These results confirm that DAG1 controls ABA and GA pathways in seeds and provide support to the notion that it promotes seed dormancy in mature dry seeds acting on these two hormones.

**Fig. 3:**
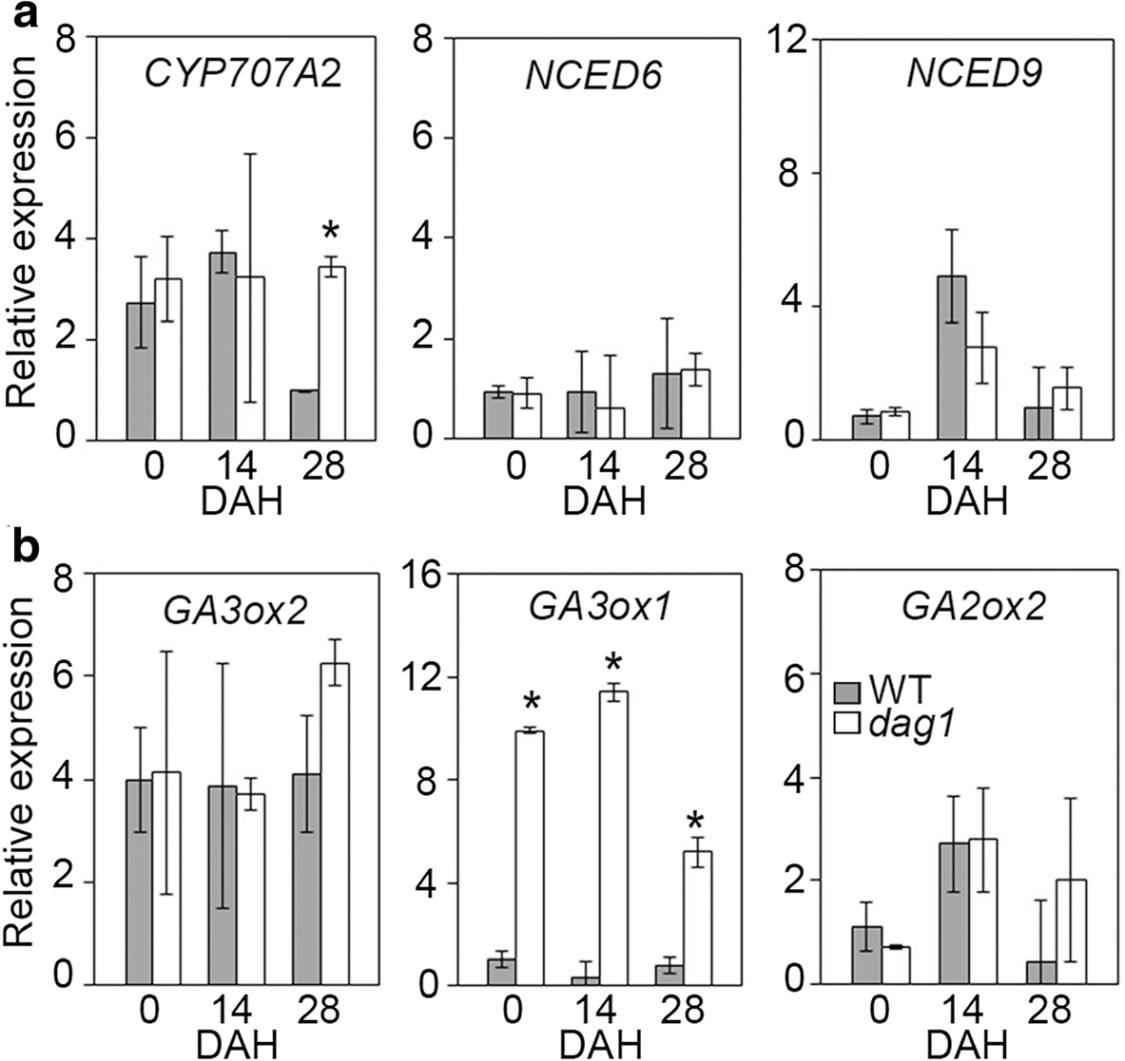
DAG1 controls ABA and GA metabolism during seed storage. Relative expression level of: **a** *CYP707A2*, *NCED6* and *NCED9*; **b** *GA3ox1*, *GA3ox2* and *GA2ox2*, in *dag1* and WT dry seeds at 0, 14 and 28 DAH. The values of relative expression levels are means of three biological replicates, presented with SD values. Expression levels were normalized with that of the *UBQ10* (*At4g05320*) reference gene. Significant differences were analyzed by *t*-test (*P ≤ 0,05)

To verify whether the post-harvest control of DAG1 on dormancy is exerted via the same hormones and genes, we compared the expression of the ABA and GAs metabolic genes in *dag1* mutant and wild type dry seeds at 0, 14 and 28 DAH. Interestingly, only the ABA catabolic gene *CYP707A2* and the GA biosynthetic gene *GA3ox1* were deregulated by *DAG1* inactivation: expression of *CYP707A2* was increased up to 4-fold at 28 DAH, while *GA3ox1* was significantly upregulated at 0, 14 and 28 DAH (Fig. 3a and b).

These results suggest that indeed DAG1 controls dormancy via the regulation of the *GA3ox1* and *CYP707A2* metabolic genes also after seed maturation and harvest, thus playing a key role in the control of the ABA/GA balance required for the developmental switch between seed dormancy and germination.

### The lack of DAG1 alters both ABA and GA levels

**Fig. 4:**
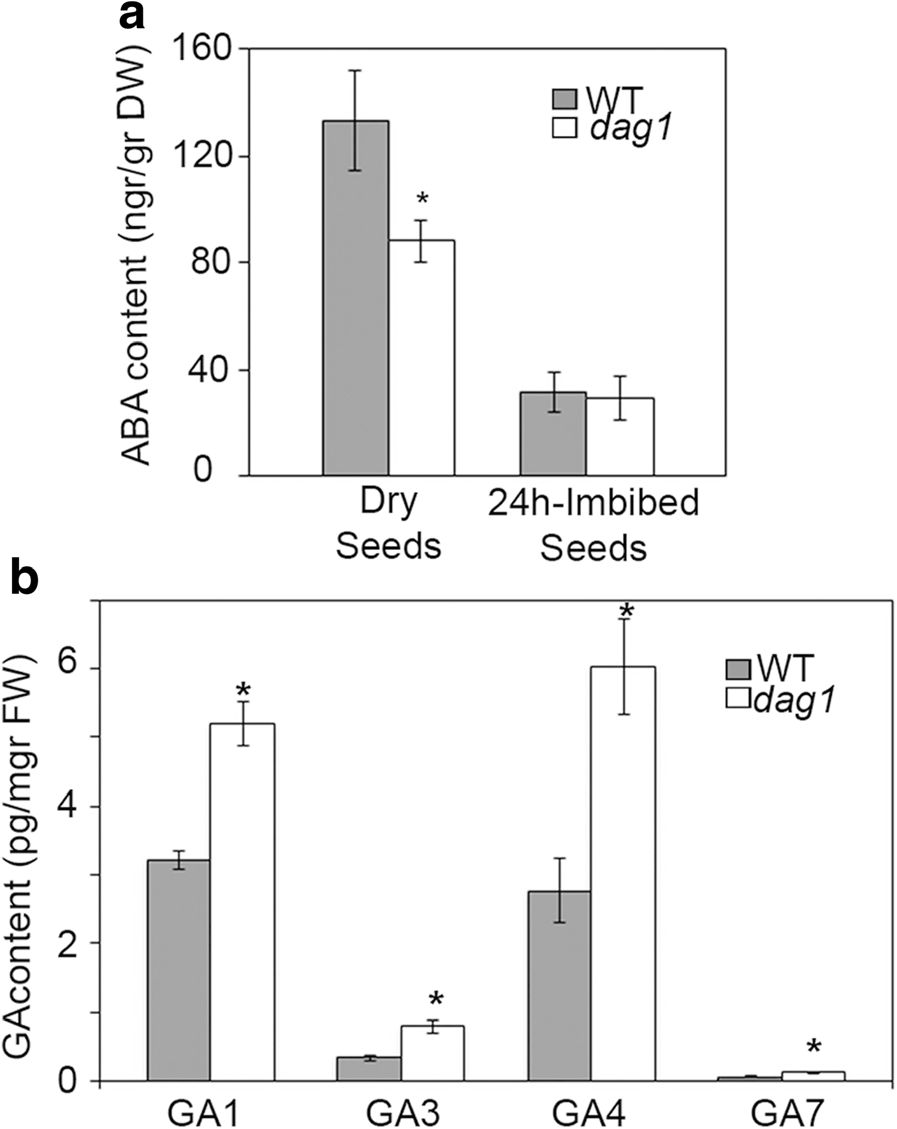
*DAG1* inactivation affects both ABA and GA levels. **a** ABA content was determined in wild type (WT) and *dag1* dry or 24 h imbibed seeds by HPLC analysis. **b** GAs (GA_1_, GA_3_, GA_4_ and GA_7_) content was determined in wild type (WT) and *dag1* 24 h imbibed seeds by UHPLC-MS/MS. The results are means of three biological replicates and are presented with SD values. Significant differences were analyzed by *t*-test (*P ≤ 0,05)

To confirm that DAG1 actually controls the levels of ABA and GAs, we measured the content of these hormones in *dag1* and wild type seeds. The amount of ABA in mature dry *dag1* seeds was significantly lower (Fig. 4a) than in wild type seeds, in agreement with the overexpression of the *CYP707A2* ABA catabolic gene. As expected, after 24 h imbibition the ABA content strongly decreased in wild type seeds [20]; interestingly, *dag1* mutant seeds showed ABA levels comparable to wild type, suggesting that the role of DAG1 in the accumulation of ABA is restricted to mature dry seeds (Fig. 4a).

Next, we measured GAs after 24 h imbibition, as in dry seeds GAs are undetectable. Consistent with the increased expression level of the GA biosynthetic gene *GA3ox1*, the amount of all bioactive GAs was significantly higher in *dag1* than in wild type seeds (Fig. 4b).

These results provide further support to the notion that DAG1 controls the levels of ABA and GAs in Arabidopsis seeds, and that the dormancy phenotype of *dag1* mutant seeds depends on alterations of both ABA and GA levels.

### DAG1 directly regulates the ABA catabolic gene *CYP707A2*

We had shown that DAG1 negatively regulates the GA biosynthetic gene *GA3ox1* by binding to its promoter [15, 16].

**Fig. 5:**
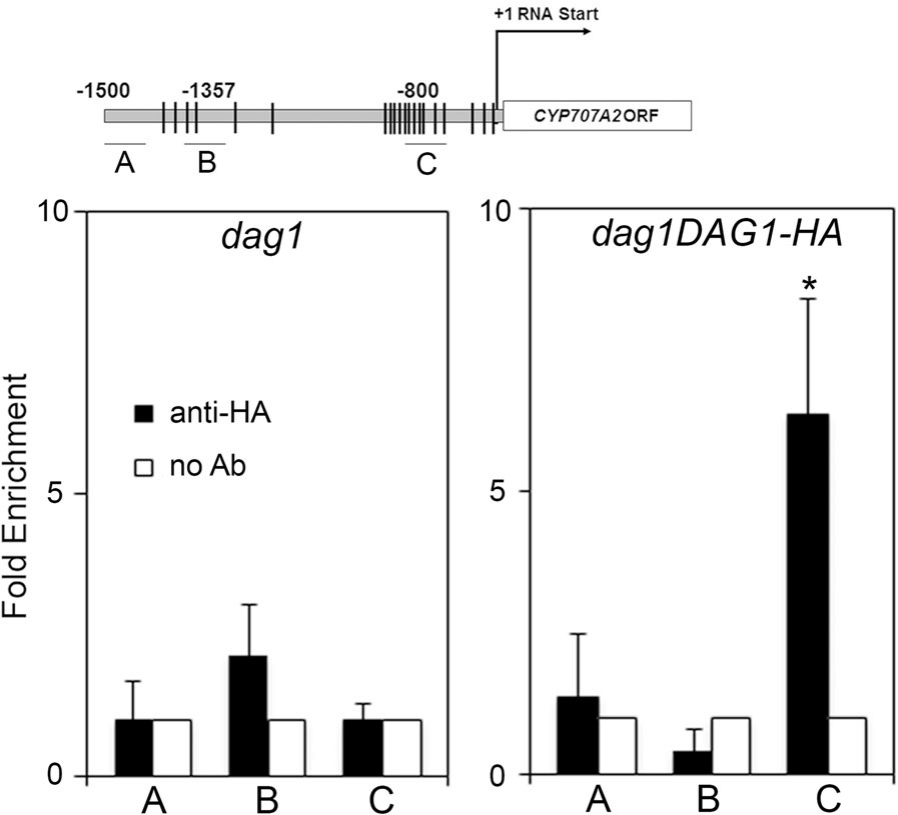
DAG1 binds the promoter of *CYP707A2. Top*: graphic representation of the *CYP707A2* promoter. Underlying thick lines marked by letters (A, B, C) are referred to different promoter fragments used for qPCR, containing 0, 2 and 10 Dof sites respectively. *Bottom*, chromatin from *dag1DAG1-HA* (*right*) seeds and from *dag1* (*left*) seeds, as a negative control, was immunoprecipitated with anti-HA antibodies, and the amount of DNA was measured by qPCR. The values of fold enrichment were normalized to internal controls (relative to input and to PP2A), and are the average of two biological replicates presented with SD values. Significant fold enrichments were analyzed by *t*-test (*P ≤ 0,05)

To assess whether DAG1 regulates also the *CYP707A2* ABA catabolic gene by directly binding to its promoter in vivo, we performed ChIP assays, using the *dag1DAG1-HA* line overexpressing the DAG1-HA chimeric protein in a *dag1* mutant background [15, 16, 21]. Cross-linked and sonicated protein–DNA complexes were precipitated with anti-HA antibodies, or without antibodies as a negative control. As additional negative control, we performed the same assays on *dag1* mutant seeds (Fig. 5, bottom left). Three regions of the *CYP707A2* promoter, one with no DOF binding sites (fragment A), one with two (fragment B), and one with ten (fragment C) were amplified by qPCR (Fig. 5, top).

The relative amount of promoter fragment C precipitated by DAG1-HA was significantly higher than the negative control, whereas the enrichment of precipitated promoter fragments A and B was very low in DAG1-HA and in the negative control (Fig. 5, bottom right), thus confirming that DAG1 directly binds to the *CYP707A2* promoter in seeds.

### GAs control *DAG1* expression and DAG1 protein stability

**Fig. 6:**
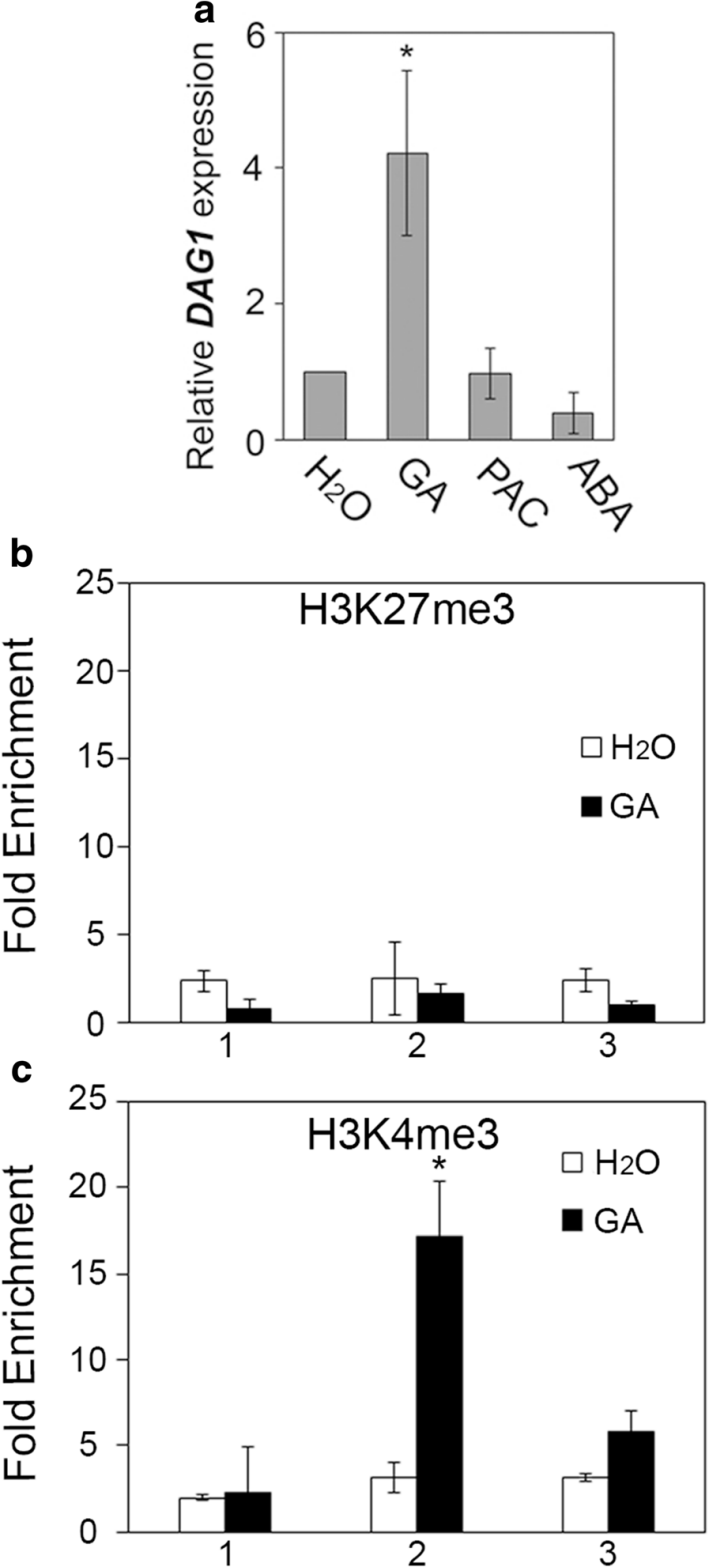
GA induces *DAG1* expression. **a** Relative expression level of *DAG1* in 24 h-imbibed wild type (WT) seeds, in the presence of water (H_2_O), GA_4+7_ (100 μM), Paclobutrazol (PAC) (100 μM), or ABA (3 μM). The values of relative expression levels are the mean of three biological replicates, presented with SD values. Expression levels were normalized with that of the *UBQ10* (*At4g05320*) gene. **b** and **c** Chromatin from seeds imbibed 24 h with H_2_O (white bars) or GA_4+7_ (100 μM) (black bars), was immunoprecipitated with specific antibodies against the H3K27me3 (**b**), or the H3K4me3 (**c**) epigenetic marks. The amount of DNA was measured by qPCR. The regions of the *DAG1* locus used for qPCR are as in Fig. 1. The values of fold enrichment were normalized to internal controls (relative to input and to PP2A), and are the average of two biological replicates presented with SD values. Significant differences were analyzed by *t*-test (*P ≤ 0,05)

Since the levels of bioactive GAs increase during seed imbibition as does the level of expression of *DAG1*, and DAG1 controls GA and ABA levels, we wondered whether *DAG1* expression in seeds might be regulated by these hormones. RT-qPCR analysis performed on seeds imbibed 24 h in the presence of GA_4+7_, or ABA, showed that the *DAG1* transcript level was significantly induced by GAs (up to 4-fold), but not by ABA. Accordingly, in the presence of paclobutrazol (PAC), an inhibitor of GA biosynthesis, *DAG1* expression level was comparable to control seeds imbibed with water (Fig. 6a).

Since we have shown above that *DAG1* is epigenetically regulated, we verified whether its induction by GAs was mediated by variations in the H3K27me3 and/or HeK4me3 epigenetic marks. This analysis, performed with wild type seeds imbibed 24 h in the presence of GA_4+7_ or with water, revealed enrichment of the H3K4me3 activating mark in GA-imbibed seeds, suggesting that *DAG1* expression is induced by GAs through chromatin remodelling (Fig. 6b and c).

**Fig. 7:**
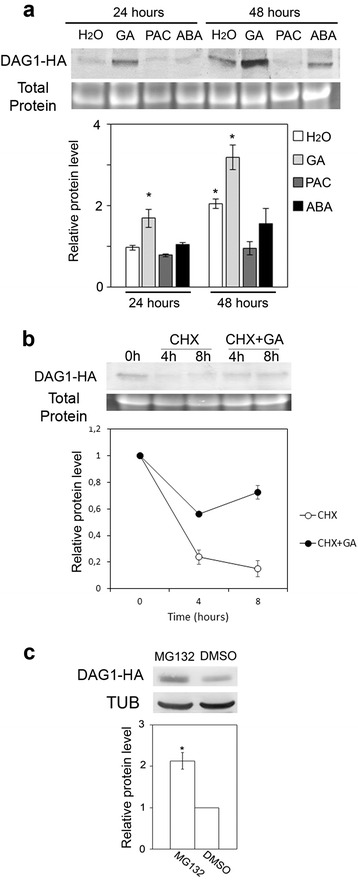
The DAG1 protein is stabilized by GA. **a** Protein level of DAG1-HA in *dag1DAG1:HA* seeds imbibed for 24 or 48 h in the presence of water (H_2_O), GA_4+7_, paclobutrazol (PAC) or ABA. **b** Protein level of DAG1-HA in 48 h-imbibed seeds (0), then treated for 4 or 8 h with cycloheximide (CHX), or with CHX and GA. Western blot (*top*) and densitometric analysis (*bottom*). **c** Protein level of DAG1-HA in 5-day-old *dag1DAG1:HA* seedlings treated with MG132 or DMSO as control. TUB or total protein content were used as loading control. Western blot (*top*) and densitometric analysis (*bottom*). GA_4+7_ (100 μM), PAC (100 μM), ABA (3 μM), CHX (50 μM), MG132 (50 μM). The protein levels are the mean of three biological replicates, presented with SD values. Significant differences were analyzed by *t*-test (*P ≤ 0,05)

To investigate whether the stability of the DAG1 protein would be affected by ABA or GAs, we utilized the *dag1* mutant line overexpressing a 35S:*DAG1-HA* translational fusion [15], whose transcription is not induced by either ABA or GAs (Additional file 2: Figure S2). We performed an immunoblot analysis on DAG1-HA seeds imbibed for 24 and 48 h in the presence of ABA, GA_4+7_ or PAC compared to water-imbibed controls. Addition of exogenous GAs increased the level of DAG1-HA, at 24 and 48 h (1.7 and 1.6-fold, respectively); consistently, the presence of PAC reduced the amount of the chimeric protein to the level of the corresponding control (Fig. 7a). Interestingly, the amount of DAG1-HA increased during imbibition up to 48 h, as a consequence of the increase of the endogenous GA levels. In contrast, the level of DAG1-HA was not affected by ABA (Fig. 7a). To gain insight on the molecular mechanism underlying this GA-mediated control of the DAG1 protein, we performed an immunoblot analysis of DAG1-HA seeds imbibed for 48 h then treated for 4/8 h with cycloheximide (CHX), to inhibit protein synthesis, or with CHX and GAs. As shown in Fig. 7b, the increase of DAG1-HA during imbibition is mainly due to new synthesis of the protein, since in the presence of CHX the amount of DAG1-HA was drastically reduced. Interestingly, addition of exogenous GAs resulted in an increase of the protein level, suggesting that GAs stabilize DAG1-HA by increasing its half-life (Fig. 7b).

We have also investigated whether the post-translational control of DAG1 by GAs is mediated by the 26S proteasome. We performed the analysis on seedlings grown for five days, then treated with MG132, a specific inhibitor of the proteasome, or DMSO as a control [22]. As shown in Fig. 7c, addition of MG132 prompted the over-accumulation of the DAG1-HA chimeric protein, suggesting that DAG1 may be negatively regulated through the 26S proteasome (Fig. 7c).

## Discussion

We had previously demonstrated that the DAG1 transcription factor is a repressor of seed germination [12, 14] acting downstream of PIL5 and negatively regulating GA biosynthesis [15, 16]. As we have also previously shown that inactivation of *DAG1* reduces seed dormancy [12], we have investigated the role of DAG1 from developing seeds to germinating seedlings.

### DAG1 controls the dynamic balance between ABA and GA

The results presented in this work suggest that DAG1 plays a key role in the establishment and maintenance of dormancy via the control of the dynamic balance of the levels of the hormones GAs and ABA.

Indeed, during seed maturation DAG1 positively regulates the ABA level by directly repressing the catabolic gene *CYP707A2*. It has been shown that CYP707A2 is a key enzyme involved in the regulation of ABA level during seed dormancy induction; in particular, expression of *CYP707A2* increases from late-maturation stage (16 DAP) to dormant seeds [20], and, consistently, *cyp707a2* null mutant seeds accumulate more ABA than wild type seeds at these stages, showing an hyperdormant phenotype [23]. These results are compatible with a role of DAG1 in promoting the establishment of dormancy during seed maturation via the negative regulation of *CYP707A2*, thus allowing an increase of ABA level before seed desiccation. Indeed, we have previously reported that in germinating seeds *CYP707A2* is upregulated in the loss-of-function *dag1* mutant [15]; consistently, *DAG1* and *CYP707A2* are both expressed in the provascular tissue of the embryo [20, 21].

It was previously shown that during dry seed storage GA levels must be maintained low to ensure seed dormancy [19, 24]; our data point to DAG1 playing a crucial role in maintaining GA biosynthesis low by repressing the expression of *GA3ox1* [15] (this work). In turn, both DAG1 expression and protein stability are controlled by GA, as the *DAG1* transcript level is increased and the protein is stabilized by bioactive GAs. This suggests that DAG1 might play a pivotal role in fine-tuning GA levels in seed germination. It has been shown that the transcript level of the GA biosynthetic gene *GA3ox1* is under feedback control by GAs [25–27]: possibly, this occurs via the increase in DAG1 level, which directly represses *GA3ox1*.

In addition, GAs promote DELLA protein degradation, and in turn they promote transcription of *DELLA* genes [28]. Interestingly, the DELLA protein GAI cooperates with DAG1 in negatively regulating *GA3ox1*, and it directly interacts with DAG1 [16]. Indeed, GAI is degraded in the presence of GAs, but it is in turn actively *de novo* synthesised in order to ensure *GA3ox1* repression through interaction with DAG1. In addition, DAG1 and GAI mutually affect their expression [16]. This regulatory loop, where GAs promote both the transcriptional induction and stabilization of a GA biosynthesis repressor (DAG1), as well as the induction of a GA signalling repressor (GAI) helps preventing early germination and/or vivipary, as well as germination under unfavorable conditions.

Similar results have been recently described in the case of the transcription factor ABI4, which controls seed dormancy by regulating the ABA and GA biosynthesis [29]. *ABI4* inactivation affects different ABA and GA metabolic genes - notably *NCED2* and *NCED3* and *GA3ox1* and *GA2ox8* respectively - and it has been shown to directly regulate the ABA catabolic genes *CYP707A1* and *CYP707A2* [29]*.* However, the effect of (the lack of) ABI4 on the expression of these genes seems to be restricted to the first six hours of seed imbibition, whereas inactivation of *DAG1* results in upregulation of *CYP707A2* and *GA3ox1* during seed maturation, storage and in seeds imbibed 12 and 24 h [15] (this work), suggesting that these two transcription factors do not function jointly. In addition, *abi4* seeds do not show reduced dormancy since the germination rate of wild type and mutant seeds were similar, although *abi4* seeds germinated more quickly than wild type and, differently from *dag1* seeds, they did not show increased sensivity to cold treatment [29], suggesting that DAG1 and ABI4 do not function in the same signaling pathway.

Our results point to DAG1 as a key regulator in the control of the developmental switch between seed dormancy and germination, acting on the balance between ABA and GAs.

### *DAG1* is controlled at the epigenetic level during seed development and early seedling growth

Our results show that trimethylation of histone H3 lysine 27 (H3K27me3), an important epigenetic mark, targets the *DAG1* locus. This chromatin repressive state is catalyzed by the Polycomb Repressive Complex 2 (PRC2), which has been shown to control the transition from seed to seedling in Arabidopsis [11].

Indeed, several seed developmental regulatory genes like *DOG1, ABSCISIC ACID INSENSITIVE 3* (*ABI3*) and *SOMNUS* (*SOM*) have been shown to be targets of PRC2 and marked by H3K27me3 during this developmental switch. In addition, Bouyer et al. [11] performed a genome-wide analysis of the chromatin state of *fie* (*fertilization independent endosperm*) mutant plants lacking PRC2, and found that *DAG1* expression is upregulated, suggesting that also *DAG1* may be a target of PRC2.

Consistently, our ChIP assays indicate that the transcribed region of *DAG1* is significantly enriched in the H3K27me3 repressive mark in seedlings, pointing to *DAG1* as a seed developmental gene.

Similarly to *DAG1*, expression of *SOM*, encoding a CCCH-type zinc finger protein, is up-regulated in *fie* mutants [11]. Interestingly, SOM down-regulates GA and upregulates ABA levels [30] to repress seed germination at high temperature [30, 31]. Our findings that *DAG1* is marked by H3K27me3 during the transition from dormant seeds to vegetative growth, further substantiate the idea that PRC2 functions during seed development to sustain the opposite action of ABA and GA as previously suggested by Bouyer et al. [11].

By means of a *pDAG1::GUS* line, we have previously shown that the *DAG1* promoter is active during embryogenesis, from globular stage to mature embryo [21]. The expression analysis we presented in this paper, performed by RT-qPCR, clearly revealed that *DAG1* is finely modulated during seed maturation and dormancy. The *DAG1* transcript level, is high at 13 DAP, it progressively decreases during maturation and dormancy and raises again in non-dormant seeds following imbibition. In addition, *DAG1* expression is induced by exogenous GAs in imbibed seeds, as the treatment with PAC results in *DAG1* expression level comparable to the control imbibed with water. Consistently, the *DAG1* locus is enriched in the H3K4me3 activating mark, as revealed by the ChIP assay performed with H3K4me3 antibodies, suggesting this epigenetic mark is necessary to ensure an active transcriptional state of the *DAG1* locus.

It was recently shown that other seed development regulatory genes such as *ABI3* and *DOG1* display a similar expression profile as *DAG1*, in that they are switched from an active to a repressive chromatin state during the transition from seed to seedling [32]. It will be interesting to assess whether all these regulatory genes work in the same or in different but possibly cross-talking regulatory networks in controlling the seed-to-seedling transition.

## Conclusions

While the importance of the balance between ABA and GAs during seed germination is well established, evidence on the molecular mechanisms underlying the transition from seed to germinating seedling is scanty. This work identifies a key component of the molecular network controlling the seed-to-seedling transition. Indeed, our work provides convincing evidence that DAG1 plays a crucial role in the establishment and maintenance of dormancy by controlling the balance of the levels of the hormones GA and ABA, acting on their biosynthesis and catabolism, respectively.

## Methods

### Plant material and growth conditions

*dag1* is the allele described in Papi et al. [12] in Ws-4 ecotype, *dag1DAG1-HA* is the transgenic line described in Gabriele et al. [15]. All *Arabidopsis thaliana* lines used in this work were grown in a growth chamber at 24/21 °C with 16/8-h day/night cycles and light intensity of 300 μmol/m^-2^ s^−1^ as previously described [12].

### Seed germination assay

All seeds used for germination tests were harvested from mature plants grown at the same time, in the same conditions, and stored for 4–5 weeks in the dark under dry conditions at room temperature. For seed germination assays, triplicate sets of 60–100 non-sterilized seeds for each genotype were sown on five layers of filter paper 595 (Schleicher & Schüll, Dassel, Germany), soaked with 5 ml water, under dim-green safe light. All germination assays have been performed with different seed batches.

### Expression analysis

RNA was extracted from developing seeds dissected from siliques at 13, 16, 19 and 21 days after pollination (DAP), dry seeds at 0, 14 and 28 days after harvest (DAH) dry seeds or imbibed seeds. The seeds were imbibed for 6, 12 or 24 h on five layers of filter paper, soaked with 5 ml water and exposed to light. For hormone treatments, seeds were imbibed for 24 h in the presence of 100 μm GA 4 + 7 (Duchefa) or 100 μm PAC (Duchefa) or 3 μm ABA (Duchefa) and exposed to light. RNA extraction and RT-qPCR were performed according to Gabriele et al. [15]. Relative expression levels were normalized with appropriated reference genes. The primers used are listed in (Additional file 3: Table S1). The values of relative expression levels are the mean of three biological replicates presented with SD values. Significative differences were analyzed by *t*-test (*P ≤ 0,05; **P ≤ 0,01).

### Chromatin Immunoprecipitation (ChIP) assay

ChIP assay was performed according to Gabriele et al. [15]. To study the binding of DAG1 to *CYP707A2*, ChIP assay was performed with the transgenic line overexpressing the DAG1-HA chimeric protein in a *dag1* mutant background and with the *dag1* mutant as a negative control. The immunoprecipitation was performed using HA-probe antibody (Y-11, sc-805 Santa Cruz). To analyse the epigenetic profile of the *DAG1* locus, chromatin was immunoprecipitated overnight using antibodies against H3K27me3 (Millipore #07-449), H3K4me3 (Abcam ab8580), or without antibodies as negative control. After reverse cross-linking, the enriched DNA levels were quantified by qPCR using specific primer sets (Additional file 3: Table S1). The Fold enrichment of a specific region was calculated respect to the internal control (*PP2A* gene) and normalized for the Input fraction, to minimize the background differences among the sample. The values are the average of two (for *CYP707A2* promoter), or three (for *DAG1* locus) biological replicates presented with SD values. Significant fold enrichments were analyzed by *t*-test (*P ≤ 0,05).

### ABA and GA dosages

Samples were analysed for GA content according to [33] with some modifications. Seed samples (20–30 mg dry weight) were homogenized in 2 ml polypropylene tubes with 1 ml of 80 % (v/v) acetonitrile containing 5 % (v/v) formic acid and 19 internal GA standards ([^2^H_2_]GA1, [^2^H_2_]GA_3_, [^2^H_2_]GA_4_, [^2^H_2_]GA_5_, [^2^H_2_]GA_6_, [^2^H_2_]GA_7_, [^2^H_2_]GA_8_, [^2^H_2_]GA_9_, [^2^H_2_]GA_12_, [^2^H_2_]GA_12_ald, [^2^H_2_]GA_15_, [^2^H_2_]GA_19_, [^2^H_2_]GA_20_, [^2^H_2_]GA_24_, [^2^H_2_]GA_29_, [^2^H_2_]GA_34_, [^2^H_2_]GA_44_, [^2^H_2_]GA_51_ and [^2^H_2_]GA_53_) (OlChemIm, Olomouc, Czech Republic) using a tissue homogenizer Precellys 24 (Bertin Technologies) at a frequency of 2x6500 rpm Hz for 2x20 s after adding 2.8 and 1.4 mm zirconium oxide beads to each tube to increase the extraction efficiency. The tubes were then placed in a 4 °C fridge and extracted overnight with constant stirring at a frequency of 15 rpm. The homogenates were centrifuged for 10 min at 4 °C. Supernatants were further purified using mixed-mode anion exchange cartridges (Waters, http://www.waters.com) and analysed by ultra high-performance chromatography (Acquity UPLC™ System; Waters) coupled to triple-stage quadrupole mass spectrometer (Xevo® TQ MS; Waters) equipped with an electrospray ionization (ESI) interface. GAs were detected using the multiple-reaction monitoring mode based on transition of the precursor ion [M-H]^–^ to the appropriate product ion. Data were acquired and processed by Masslynx 4.1 software (Waters) and GA levels were calculated using the standard isotope-dilution method [34]. For ABA dosage, seeds were frozen in liquid nitrogen and freeze-dried. The dried seeds (10 mg) were ground in 1.6 ml of extraction solvent (acetone/water/acetic acid, 80/19/1, v/v/v), in which 2 ng of [^2^H_4_]ABA ((-)-5, 8′, 8′, 8′-d4 ABA purchased from Irina Zaharia, Plant Biotechnology Institute, National Research Council Canada, http://www.nrc-cnrc.gc.ca) was added as an internal standard. Samples were centrifuged and the supernatant recovered, the pellet was then re-suspended in 0.1 ml of chromatography mobile phase by sonication, re-centrifuged and the supernatants combined. The extraction solvent was then evaporated and the residue re-suspended by sonication in 0.5 ml of chromatography mobile phase (acetonitrile/water/acetic acid, 50/50/0.05, v/v/v) and filtered through a 1.6 μm GFA filter (Whatman, http://www.whatman.com/). ABA was quantified using a LC-ESI-MS-MS system (Quattro LC, Waters, http://www.waters.com) in a positive ion mode by multiple reaction monitoring (MRM). The results are means of two (ABA dosages) or three (GA dosages) biological replicates and are presented with SD values. Significant differences were analyzed by *t*-test (*P ≤ 0,05).

### Immunoblot analysis

A total of 25 μg of protein was extracted according to Oh et al. [35] and separated on a 12 % SDS-polyacrylamide gel (Bio-Rad) and blotted on a PVDF Immobilon-P Transfer membrane (Millipore). Detection of chimeric proteins was performed with anti-HA antibodies (Santa Cruz, Santa Cruz, CA, USA) as primary antibody and AP-conjugated anti-mouse as secondary antibody (Sigma, St. Louis, U.S.A.). Total proteins revealed by stain-free technology (Bio-Rad), or tubulin levels detected using an anti-tubulin antibody (Sigma, St. Louis, U.S.A.) were used as loading control. For MG132 treatment, 5-days-old dark grown seedlings were treated 4 h with MG132 (50 μm) or DMSO in dark or R light. For GA, Paclobutrazol (PAC) or ABA treatments, 50 μl seeds were imbibed 24 or 48 h, in the presence of 100 μm GA _4+7_ (Duchefa) or 100 μm PAC (Duchefa) or 3 μm ABA (Duchefa). For CHX treatment, 50 μl seeds were imbibed 24 h in the presence of GA, or 48 h with water, then transferred on CHX (50 μm) for 4 or 8 h. The protein levels are the mean of three biological replicates, presented with SD values. Significant differences were analyzed by *t*-test (*P ≤ 0,05).

## Additional files

Additional file 1: Figure S1. Negative controls of the ChIP assays. Chromatin from WT embryos at 10/13 DAP, 0 DAH, 24 h-imbibed seeds, and 14 days-old seedlings was immunoprecipitated without antibody as a negative control. The amount of DNA was measured by qPCR. The values of fold enrichment were normalized to internal controls (relative to input and to *PP2A*), and are the average of three independent experiments presented with SD values. (PDF 265 kb) Additional file 2: Figure S2. Relative expression level of *35S::DAG1-HA*, in imbibed seeds. Relative expression level of DAG1-HA, under the control of the 35S CaMV promoter, in *dag1DAG1-HA* seeds, imbibed 24 or 48 h. The values of relative expression levels are the mean of three biological replicates, presented with SD values. Expression levels were normalized with that of the *UBQ10* (At4g05320) gene. (PDF 275 kb) Additional file 3: Table S1. Primers used in this study. (PDF 554 kb)

## Abbreviations

ABA: Abscisic Acid
GA: Gibberellins
H3K27me3: Histone H3 at lysine 27 trimethylated
H3K4me3: Histone H3 at lysine 4 trimethylated
DAG1: DOF AFFECTING GERMINATION1
GA3ox1: Gibberellin 3-β-dioxygenase 1
DOG1: DELAY OF GERMINATION1
QTL: Quantitative Trait Loci
PRC2: Polycomb Repressive Complex 2
phyB: Phytochrome B
PIL5: PHYTOCHROME INTERACTING FACTOR3 LIKE5
GAI: GA INSENSITIVE
DAP: Days after pollination
DAH: Days after harvest
ChIP: Chromatin Immuno Precipitation
RT-qPCR: Quantitative reverse transcriptase-polymerase chain reaction
qPCR: quantitative PCR
PAC: Paclobutrazol
CHX: Cycloheximide
DMSO: Dimethyl sulfoxide
fie: Fertilization independent endosperm
ABI3: ABSCISIC ACID INSENSITIVE 3
SOM: SOMNUS
