# Supplementary material for: The DAG1 transcription factor negatively regulates the seed-to-seedling transition in Arabidopsis acting on ABA and GA levels
Source: BMC Plant Biol. 2016 Sep 9;16(1):198. doi: 10.1186/s12870-016-0890-5 (PMC5016951; doi:10.1186/s12870-016-0890-5)
Supplement: Additional file 1: Figure S1. — Negative controls of the ChIP assays. Chromatin from WT embryos at 10/13 DAP, 0 DAH, 24 h-imbibed seeds, and 14 days-old seedlings was immunoprecipitated without antibody as a negative control. The amount of DNA was measured by qPCR. The values of fold enrichment were normalized to internal controls (relative to input and to PP2A), and are the average of three independent experiments presented with SD values. (PDF 265 kb) [file 12870_2016_890_MOESM1_ESM.pdf]

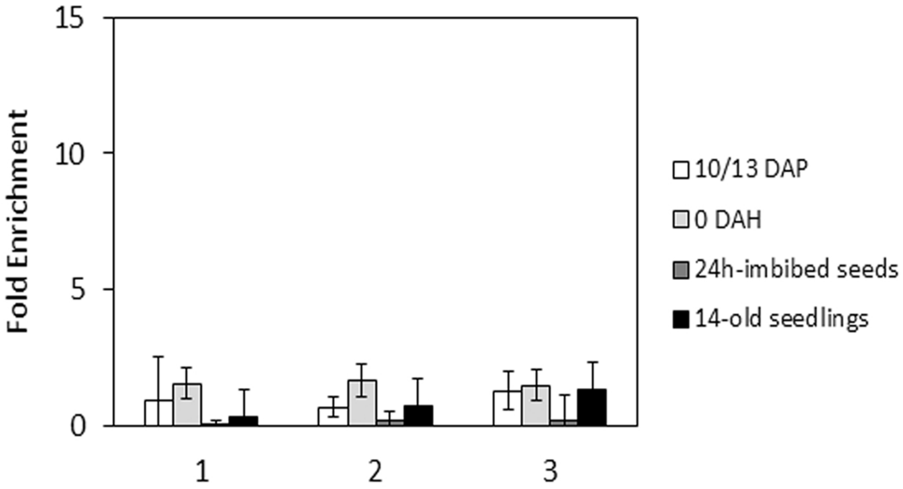

Fig. S1 Negative control of the ChIP assays.
Chromatin from WT embryos at 10/13 DAP, 0 DAH, 24 hours-imbibed seeds, and 14 days-old seedlings was immunoprecipitated without antibody as a negative control. The amount of DNA was measured by qPCR. The values of fold enrichment were normalized to internal controls (relative to input and to *PP2A*), and are the average of three independent experiments presented with SD values.
