# Supplementary material for: The DAG1 transcription factor negatively regulates the seed-to-seedling transition in Arabidopsis acting on ABA and GA levels
Source: BMC Plant Biol. 2016 Sep 9;16(1):198. doi: 10.1186/s12870-016-0890-5 (PMC5016951; doi:10.1186/s12870-016-0890-5)
Supplement: Additional file 2: Figure S2. — Relative expression level of 35S::DAG1-HA, in imbibed seeds. Relative expression level of DAG1-HA, under the control of the 35S CaMV promoter, in dag1DAG1-HA seeds, imbibed 24 or 48 h. The values of relative expression levels are the mean of three biological replicates, presented with SD values. Expression levels were normalized with that of the UBQ10 (At4g05320) gene. (PDF 275 kb) [file 12870_2016_890_MOESM2_ESM.pdf]

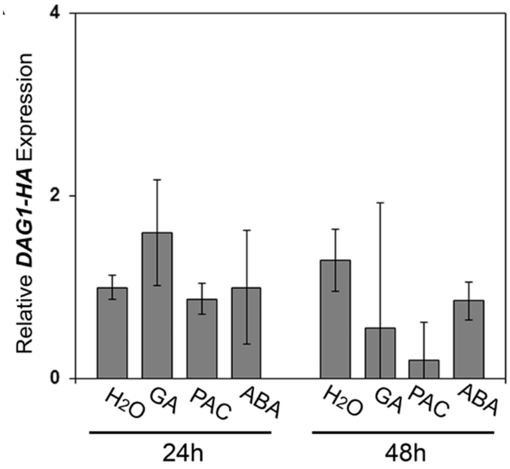

Fig. S2 Relative expression level of *35S::DAG1-HA*, in imbibed seeds
Relative expression level of *DAG1-HA*, under the control of the 35S CaMV promoter, in *dag1DAG1-HA* seeds, imbibed 24 or 48 hours. The values of relative expression levels are the mean of three biological replicates, presented with SD values. Expression levels were normalized with that of the *UBQ10* (*At4g05320*) gene.
