# Supplementary material for: The DAG1 transcription factor negatively regulates the seed-to-seedling transition in Arabidopsis acting on ABA and GA levels
Source: BMC Plant Biol. 2016 Sep 9;16(1):198. doi: 10.1186/s12870-016-0890-5 (PMC5016951; doi:10.1186/s12870-016-0890-5)
Supplement: Additional file 3: Table S1. — Primers used in this study. (PDF 554 kb) [file 12870_2016_890_MOESM3_ESM.pdf]

Table S1. Primers

ChIP-qPCR primers	Forward (5'-3')	Reverse (5'-3')
***pCYP707A2*-a**	GTCCGAATCCAACTTGTGCT	ATGTTCTCCAAGGCCCAATA
***pCYP707A2*-b**	AGTGAAGAGGTAGCCGTAGCA	TCGTTGATGATGGTTAGTGTCC
***pCYP707A2*-c**	AAGGGTTGGAGCATGTTTTG	TCCCCGATTTGTCTCTGTCT
***DAG1 promoter* 1**	CAAAACGACACGAAATGTGG	GAGATAGGCCCCAGTTTTGA
DAG1 5'UTR 2	GAGAGCCATATCGTCCCAAA	CCACTTCGTAGCATCCATGA
DAG1 gene 3	CGCAACAACAACCAACATTC	GCCGTGTTGTTGGTATTTCC
PP2A	TGCTGAAGAGTTGGTCCTG	GAAGCGATACTGCACGAAGA

RT-qPCR primers	Forward (5'-3')	Reverse (5'-3')
CYP707A2	ATGGGGTTGCCTTACATCGGAGA	TGGCTTGAACAAGTGAGCTTTGCT
NCED6	ACCGGGTCGGATATAAATTGGGTTG	CCCGGGTTGGTTCTCCTGATTC
NCED9	AACCGCCGCTATGGTTTTAGACG	CCAGTCACCGGAAGGTTATGCAC
GA3ox1	GCTTAAGTCTGCTCGGTCGG	AGTGCGATACGAGCGACG
GA3ox2	ACGTCGGTGACTTGCTCCA	GTTAACCCTGGCTCGGTGAA
GA2ox2	TCCGACCCGAACTCATGACT	CGGCCCGGTTTTTAAGAGAC
DAG1	TTGTCGAAGGTATTGGACCGA	CCGACTGGGACGTTACGAAG
UBQ10	GGCCTTGTATAATCCTGATGAATAAG	AAAGAGATAACAGACGGAAACATAGT
